# Visual order of Chinese ink paintings

**DOI:** 10.1186/s42492-020-00059-5

**Published:** 2020-10-12

**Authors:** Zhen-Bao Fan, Kang Zhang

**Affiliations:** Department of Computer Science, The University of Texas at Dallas, Richardson, TX 75082-3021 USA

**Keywords:** Aesthetic measurement, Paintings, Visual order, Visual information processing

## Abstract

Visual order is one of the key factors influencing the aesthetic judgment of artworks. This paper reports the results of evaluating the influence of extracted features on visual order in Chinese ink paintings, using a regression model. We use nine contemporary artists’ paintings as examples and extract features related to the visual order of their paintings. A questionnaire survey is conducted to collect people’s rating scores on the visual order. Via regression modeling, our research analyzes the significance of each feature and validates the influences of the features on the visual order.

## Introduction

**Fig. 1:**
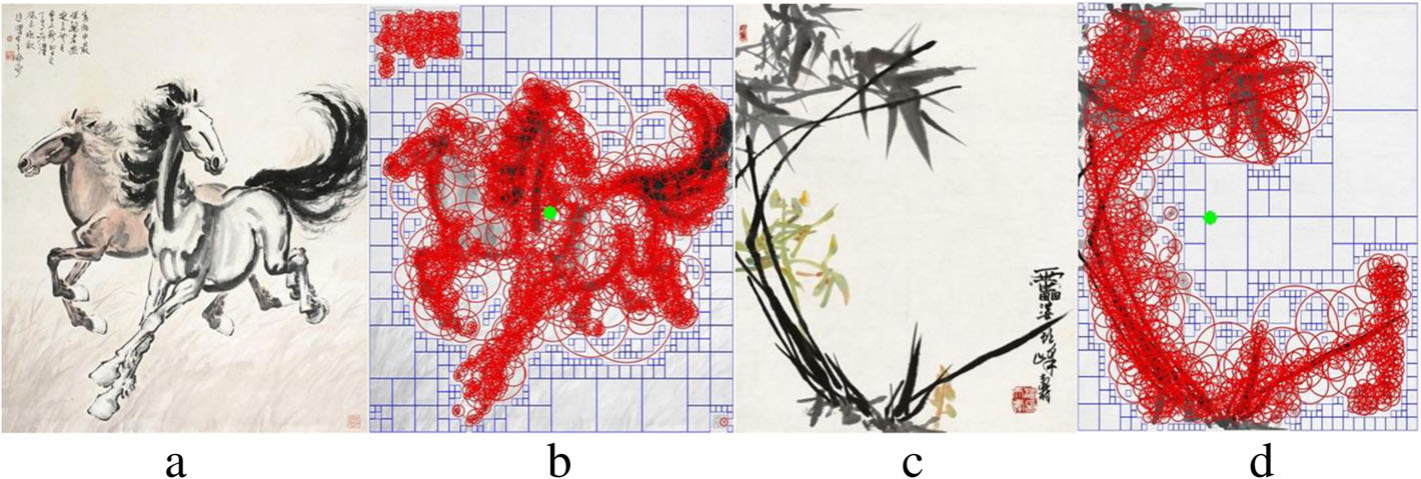
Comparison of two paintings with local symmetry marked by red circles, white space marked by blue lines and mass center marked by filled green circles. **a** Two Horses; **b** Rating score of visual order on Two Horses: 5.94, local symmetry (gradient): 86, white space: 44.1%, mass center: − 0.0034, 0.0129; **c** Orchid and Bamboo; **d** Rating score of visual order on Orchid and Bamboo: 5.37, local symmetry (gradient): 202, white space: 33.6%, mass center: − 0.1101, − 0.0017

Visual order refers to the arrangement of elements according to similarity and difference, repetition and symmetry in the elements. It exists in many types of forms, from a string of beads to the layout of a page, speech and music to structures of society and even the systems of thought. In this paper, we focus on the order of artworks, specifically, the visual order of Chinese ink paintings. We attempt to explore the quantified features influencing the visual order of Chinese ink paintings, including local symmetry, white space and mass center. Figure 1 shows the quantified results of two of the selected paintings, which will be fully explained in Methods. The coefficients and correlation values collected from the linear regression model are used to evaluate the influence of the features.

### Motivation

Artwork digitalization and web publishing make it possible for the general population to view artworks online. One could appreciate artworks at home through online art libraries or galleries without going to museums. This trend is becoming obvious and accelerated during the current pandemic. If the aesthetic quality of online artworks could be digitally evaluated, art management and artwork recommendations would become more effective and objective digitized online platforms. Artists could also have one more assisting tool to evaluate how their works are appreciated by viewers. There is, however, little research in the aesthetic quality assessment of artworks. Birkhoff’s aesthetic measure is an art theory that simplifies the assessment as a combination of complexity and order. The sense of order is commonly related to beauty. This work attempts to discover whether one could map artworks’ beauty to the mathematical forms of feature calculations.

In the previous theoretical studies on visual processing and perception, researchers have proposed different approaches for quantitative measurement of websites, digital photographs and images of varied qualities. For example, in the field of image compression, metrics have been proposed to evaluate the visual quality of compressed images/videos and obtain conclusions in accordance with human’s evaluations. Researchers also attempt to understand the human’s visual and aesthetic perception of a Web page and the relation between the aesthetic appearance of a Web page and its visual complexity.

Photographs and paintings are similar in many ways. For example, a realistic oil painting may look like a photograph at first glance. Existing literature on aesthetic/quality assessment of photographs and paintings mostly use classification or regression techniques, such as classifying photographs as professional or snapshots, classifying paintings as high quality or low quality, or predicting aesthetic rating scores. But the perception and evaluation of paintings are different from photographs. Paintings have various genres, such as, representational vs abstract painting, Mondrian’s abstract style vs Picasso’s cubism style, with totally different patterns and visual element. Therefore, paintings cannot be simply put together for aesthetic assessment as photographs.

Because the variance among human ratings on paintings can be large, in this paper, we simplify and enhance the feasibility of our study in the following aspect. We focus on one type of paintings, Chinese ink paintings, and select the paintings that can narrow the variance in styles and contents. Chinese ink paintings are distinguished from paintings of other genres. For example, traditional oil paintings focus on complete realism and details, but Chinese ink paintings pay more attention to artistic conception and expressiveness. They also demonstrate the exquisite and implicit usage of white space. We select paintings of nine contemporary artists, who share similar styles, contents and techniques.

Our research provides a quantitative method to describe visual order of Chinese ink paintings and contributes to the existing literature by:

- Proposing computational methods to gauge the features influencing the visual order of Chinese ink paintings;
- Highlighting an objective view to understand humans’ perception of visual order;
- Validating the influence of visual features on visual order via an empirical study.

We review the aesthetic theory established over the years, the existing works of aesthetic quality assessment, and previous studies on order.

### Aesthetic theory in philosophy and art

The topic of beauty has been debated for at least 2500 years and has attracted a wide variety of interpretations [1]. These interpretations can be broadly divided into three categories [2]: the objective view (beauty as object producing a pleasurable experience for the viewer), the subjective view (beauty can be anything if it pleases the sense of the beholder) and the interactionist perspective (beauty emerges from patterns in the way people and objects relate). Art theories have studied the perceptual features of artworks [3]. With respect to such aesthetic judgments, many perceptual features have been investigated, such as contrasts, visual complexity, color symmetry and grouping.

There have been much important research into aesthetic preferences. For example, researchers investigate elementary features influencing one’s aesthetic experience in viewing paintings [4], built on Birkhoff’s theory of aesthetic measure [5]. Birkhoff regards aesthetic measure (M), order (O) and complexity (C) as measurable contributors and derives the following formula: M = O/C. It groups the features, such as symmetry, contrasts, into one term, order. Visual complexity refers to the number and quantity of basic visual elements. The desire for complexity is considered an important aesthetic experience to activate the perceptual system and find regularities. The visual complexity of Chinese ink paintings can also be addressed by objective measurements of features influencing complexity [6]. This article attempts to measure visual order, related to the composition and arrangement of visual elements, influenced by the rules of harmony, symmetry and balance, etc.

### Aesthetic quality assessment and enhancement

In computer science, aesthetic quality assessment has been attracting increasing attention. Many methods have been proposed, such as, a binary classification problem for predicting high-quality or low-quality of an image, a regression problem of producing aesthetics scores [7], a graph grammar approach [8] for evaluating automatically generated designs [9].

For example, the classification between high-quality professional photos and low-quality snapshots can be implemented by determining the perceptual features that distinguish two types of photos and designing high-level semantic features to measure the differences [10]. Global and local characteristics are used to classify oil paintings into high-quality and low-quality [11]. Image aesthetic analysis could also be formulated as a regression problem. Researchers predict the numerical aesthetics ratings on peer-rated online photographs using linear regression on the polynomial terms of features [12]. Experimental results of a regression model using convolutional neural networks show that the aesthetic assessment on photographs is similar to human visual system effectively and outperforms previous methods [13].

Apart from the works that focus on quality assessment, researchers have also investigated quality enhancement. For example, after a user interactively selects a foreground object in the photo, the system presents recommendations for where it can be moved in a manner that optimizes a learned aesthetic metric [14]. Another proposed method may enhance the harmony among the colors of a given photograph by shifting its hue values to fit the selected optimal color schema [15]. Using a deep learning-based method, another study attempts to crop an input image into an aesthetically pleasing one, while preserving its important parts as much as possible [16].

Although many works have been published on photograph assessment, little work has been done on aesthetic assessment of paintings. The methods used in photograph quality assessment like feature extraction and data analysis could, however, be potentially adapted for paintings’ aesthetic assessment.

### Visual order

Visual order offers an understanding of beauty in a form of interpretation that integrates quantitative and qualitative aspects of experience. Order relates to the arrangement of elements in terms of similarity vs difference, and repetition vs symmetry [17]. In the environmental aesthetics literature, order is defined as the degree and type of lawfulness governing the relations among the parts of an entity [18]. An ordering principle is a law, a rule, a pattern or a form by which the elements of a given set can be arranged [19]. The visual order can be found in a wide range of entities, such as landscape, architecture, artworks, etc. It is an important feature in aesthetic quality assessment. For example, visual order is a salient aesthetic feature on customers’ preferences for web pages [20]. Birkhoff defines the aesthetic measure as the order divided by complexity [5], which are two terms respectively related to characteristics of realization of elements, such as harmony, symmetry, and level of details and intricacy of elements.

In the remaining part of this paper, we first provide the details of the calculation of features and describe how we conduct the questionnaire survey. Then we evaluate the features and generate a regression model of visual order, followed by our conclusions in the last section.

## Methods

Inspired by the assessment of photographs and paintings of other genres, we investigate different features related to visual order and evaluate whether these features are influential or not.

### Image segments

Human vision is sensitive to large segments in an image. Some regions with similar color and spatially adjacent can be merged into one region. Local characteristics, such as the largest segment, include detailed information that may attract viewers’ attention [11]. Paintings can be segmented into different parts to extract local features and the first and SLSs (the second largest segments) contain important information. Using the software packages and methods, such as MarvinSegment, in Marvin Framework (http://marvinproject.sourceforge.net) to calculate the segmentation attributes [21], we extract the first largest segment (FLS), SLS and calculate the following features [22].

#### Number of segments

A painting has regions of the same color and these regions can be calculated and counted as segments. If a painting is homogenous and has a simple composition, it should have a small number of segments. In contrast, if a painting is composed of many different colors and parts, it could be segmented into many pieces. The number of segments may be relevant to visual order.

#### Area sizes of two largest segments

If a painting has a large FLS and/or SLS, it must contain at least a large homogenous region (segment). The painting has increased possibility to be flat and simpler than the one with smaller FLS and SLS.

#### Average hue, saturation and lightness of FLS and SLS

Apart from the overall color characteristics of the entire image, we also calculate the average hue, saturation, and lightness for the top two largest segments.

#### Hue, saturation, lightness contrast

We calculate the contrasts of hue, saturation and lightness between FLS and its neighboring segments. Contrast features among segments around FLS indicate the relationship between the major regions of a painting.

### Color complexity

The distributional characteristic of color stimuli is possibly related to visual order because a color image can be composed in a monochrome or polychrome manner. Different spatial distributions can give the viewer different color perception feelings.

We use color complexity measurement (CCM) [23] to measure the spatial composition of colors, i.e., how intricate color is used in a painting. The method calculates the CCM value within the local mask of each pixel and then computes the average CCM value for the entire image. A high CCM value means a high spatial pattern variation among neighboring pixels, while a small one means that the region around this pixel is homogenous.

### White space

White space is an established characteristic and a figure of visual rhetoric in Chinese ink paintings [24]. It is more than a blank space but indicates the semantic meaning of a vast, formless and ever-changing natural phenomenon, such as sky, clouds, water, etc. It is a technique widely and deliberately used in Chinese ink paintings, aiming to evoke viewers’ anticipation, activate their imagination, and invite their elaboration. Because white space is an important feature in Chinese ink paintings, it would affect visual order. We capture white space in the collected paintings by applying quadtree decomposition to them [6]. The quadtree algorithm recursively divides a painting image into four quadrates until all divided quadrates cannot be further divided when reaching a criterion. We set the criterion as whether each quadrate is filled with over 70% of white pixels. We determine a divided quadrate as a white that could be a part of white space. If a quadrate has less than 70% of white pixels, the algorithm continues to divide it into four quadrates, and finally sums the white quadrates in each painting to represent white space. Figure 1 shows white quadrates in a painting, where each white space is marked by blue lines.

### Edge density

Edge density is measured by the ratio of the pixel number of the extracted edges divided by the pixel number of the entire image [22].

### Symmetry

Symmetry is commonly seen in various structures, such as artificial shapes, architecture and man-made scenes [25]. Detected early in artworks [26], symmetry could facilitate the viewer’s processing of artworks [2] and tends to be more preferred than non-symmetry [27]. Viewers have innate abilities to perceive symmetries. The measurement of symmetry is a long-standing problem. In computer science, symmetry is a stable and robust feature in image processing. In psychology and art, it is an established stimulus. Researchers have made significant progress in representing and detecting symmetries in different types of media including images [25].

A well-designed symmetry could enhance the feeling of order in a painting. Chinese ink painting artists typically prefer an implicit way of presentation. For example, instead of directly drawing real-world objects, they commonly use white space as a rhetoric form in presenting semantic objects. Similarly, the symmetric forms in Chinese ink paintings are usually not at the big scale, e.g., the entire paintings, but rather, most often at local and smaller scales.

In this paper, we choose an established method to extract local symmetry features [28]. It computes local features based on a softer definition of symmetry score and calculates local symmetry scores over the image and across scale space. We calculate intensity-based local symmetry (or simply SYM-I) and gradient-based local symmetry (or SYM-G). The local symmetry score is computed for each pixel *p* in an image by summing the local symmetry distances between *p* and other pixels in a Gaussian mask around *p*. The symmetry distance includes two terms, the absolute difference *d* between the intensity that measures how well a pair of symmetric points match each other in appearance, and the weight *w* that measures the importance of each point pair. A lower symmetry distance indicates greater local symmetry. Red circles in Fig. 1 visualize the detected features of symmetry in different scales. A circle covers a detected symmetric region. Different sizes of circles represent different scales of symmetries.

### Mass center

Paintings may be viewed to be “well-balanced” or “poorly-balanced” and aestheticians are unanimous about the importance of balance [29]. Picture balance is considered an “indispensable factor in aesthetic composition” and could be measured [30]. Mass center is a relative position representing the balance point of the distributed mass in space. We believe that the position of mass center could influence the visual order of paintings. We transform a colored image to grayscale and calculate the mass center of the transformed image using the intensity of each pixel.

#### Coordinates of mass center

We calculate the mass center of each painting and obtain its horizontal and vertical coordinates.

#### Relative positions of mass center

Since the selected paintings have varied sizes, we also calculate the relative positions of the mass center which divides the coordinates by each painting’s width and height values.

#### Mass Center’s distance to Painting’s center

We compute the distance between the mass center and the image center in each painting. Different distance measurements are included, such as Euclidian distance and Manhattan distance. Specially in Manhattan distance, considering the different sizes of the paintings, we consider the distance as $\frac{d1}{w}+\frac{d2}{h}$. Here *d*1 and *d*2 are the distances to the middle of the painting on *x*-axis and *y*-axis respectively, and *w* and *h* are the width and height of the painting respectively.

### Survey

We present our survey for collecting order-related ratings on the selected paintings as follows.

#### Materials

In this experiment, we select Chinese ink paintings by nine contemporary Chinese artists, including Wu Guanzhong (1919–2010), Qi Baishi (1864–1957), Xu Beihong (1895–1953), Wu Hufan (1894–1968), Huang Binhong (1865–1955), Gao Jianfu (1879–1951), Liu Haisu (1896–1994), Dong Shouping (1904–1997) and Li Keran (1907–1989). These artists inherit traditional skills of Chinese ink paintings and also infiltrate modern styles. The paintings used for this experiment are collected in the online archive.

#### Procedure

**Fig. 2:**
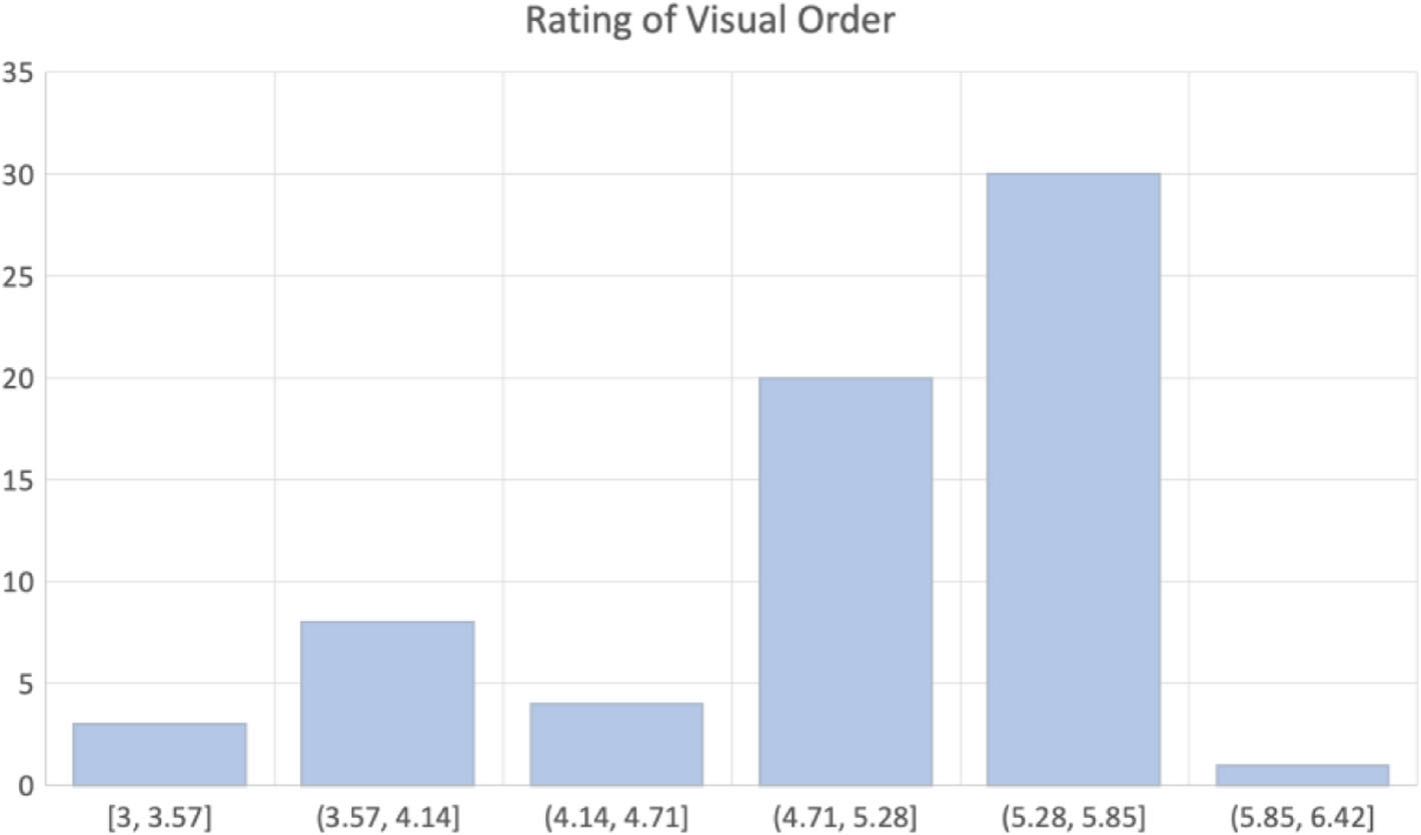
Distribution of rating scores

We conducted this experiment on a Chinese online panel. Sixty-six selected paintings are divided to make six surveys with exactly the same questions. In each survey, 50 participants were recruited and answer questions on a computer monitor. Each painting is viewed for at least 5 s without a maximum time limit [31]. After viewing each painting, they are required to give a rating on the visual order of the paintings (1 = unordered and 7 = ordered) on the 7-point scale [32]. None of the participants is reported to be color blind or an art expert. Among all the participants, 127 are male and 173 are female. We calculate the average rating score of each painting and depict the score distribution among 66 paintings in Fig. 2. No painting was given less than 3 on visual order.

## Results and discussion

We compute the average rating on the visual order of each painting and build a linear regression model using the average rating scores as a dependent variable and the calculated features as independent variables. The results in Table 1 show that white space, mass center’s relative Manhattan distance to the image center, and gradient-based local symmetry could influence the visual order. *R*^2^ represents the proportion of the variance in the dependent variable that is predictable from the independent variables. *F* is the F-statistic and *Sig*. is the *p* value of the model. Our model explains 61.0% of variances in independent variables and is thus statistically significant (Model *Sig.* < 0.05). The significance values of three features are also listed in the Significance column. All independent variables are statistically significant (*Sig.* < 0.05), which means the changes in the three features correlate to shifts in the rating score. We standardize the independent variables before running linear regression in Table 1. Table 2 lists the mean and standard deviations of three main features before standardization. The percentage of white space varies from 0.0 to 0.6 in selected paintings and the standard deviation is 0.15. But the mass center’s distance to center does not show large variance compared to white space. The mass center’s relative Manhattan distance to the image center varies from 0.005 to 0.15. The standard deviation is also smaller than white space, which implies that the mass centers are off but close to the physical centers. Table 3 shows the relationship between visual order and the computed visual features.

**Table 1:** Linear regression model predicting the paintings’ visual order

Model Summary			
*R*^*2*^ = 0.627 adj. *R*^*2*^ = 0. 609 F = 34.694 Sig. = 0.00			
Variable	Regression coefficients	Standard error	Significance
White space	0.234	0.056	0.000
Mass Center’s Distance to Center	0.163	0.054	0.004
SYM-G	−0.311	0.059	0.000
Constant	4.996	0.051	0.000

*R*^*2*^ R-Squared, *adj. R*^*2*^ adjusted R-Squared, *F* F-statistic, *Sig*. model significance

**Table 2:** Statistics of three main features

	Mean	Std. Deviation	Minimum	Maximum
White space	0.18	0.15	0.00	0.60
SYM-G	143.47	129.20	8.74	662.28
Mass Center’s Distance to Center	0.07	0.04	0.005	0.15

**Table 3:** Correlation between the rating of visual order and computed features

	Pearson correlation	Sig.
Number of segments	−0.006	0.964
Area size of FLS	−0.078	0.534
Area size of SLS	0.005	0.97
Hue of FLS	0.19	0.127
Saturation of FLS	0.163	0.192
Lightness of FLS	0.057	0.647
Hue of SLS	−0.054	0.665
Saturation of SLS	0.107	0.394
Lightness of SLS	0.01	0.933
Hue contrast	0.349	0.005
Saturation contrast	0.133	0.294
Lightness contrast	0.04	0.755
Color complexity	−0.513	0.000
White space	0.568	0.000
Edge density	−0.449	0.000
SYM-G	−0.685	0.000
SYM-I	−0.517	0.000
*x*-axis of mass center	−0.598	0.000
*y*-axis of mass center	0.395	0.001
Relative *x*-axis of mass center	0.081	0.517
Relative* y*-axis of mass center	0.309	0.011
Euclidian distance to center	0.365	0.003
Manhattan distance to center	0.356	0.003
Relative Manhattan distance to center	0.429	0.000

**Fig. 3:**
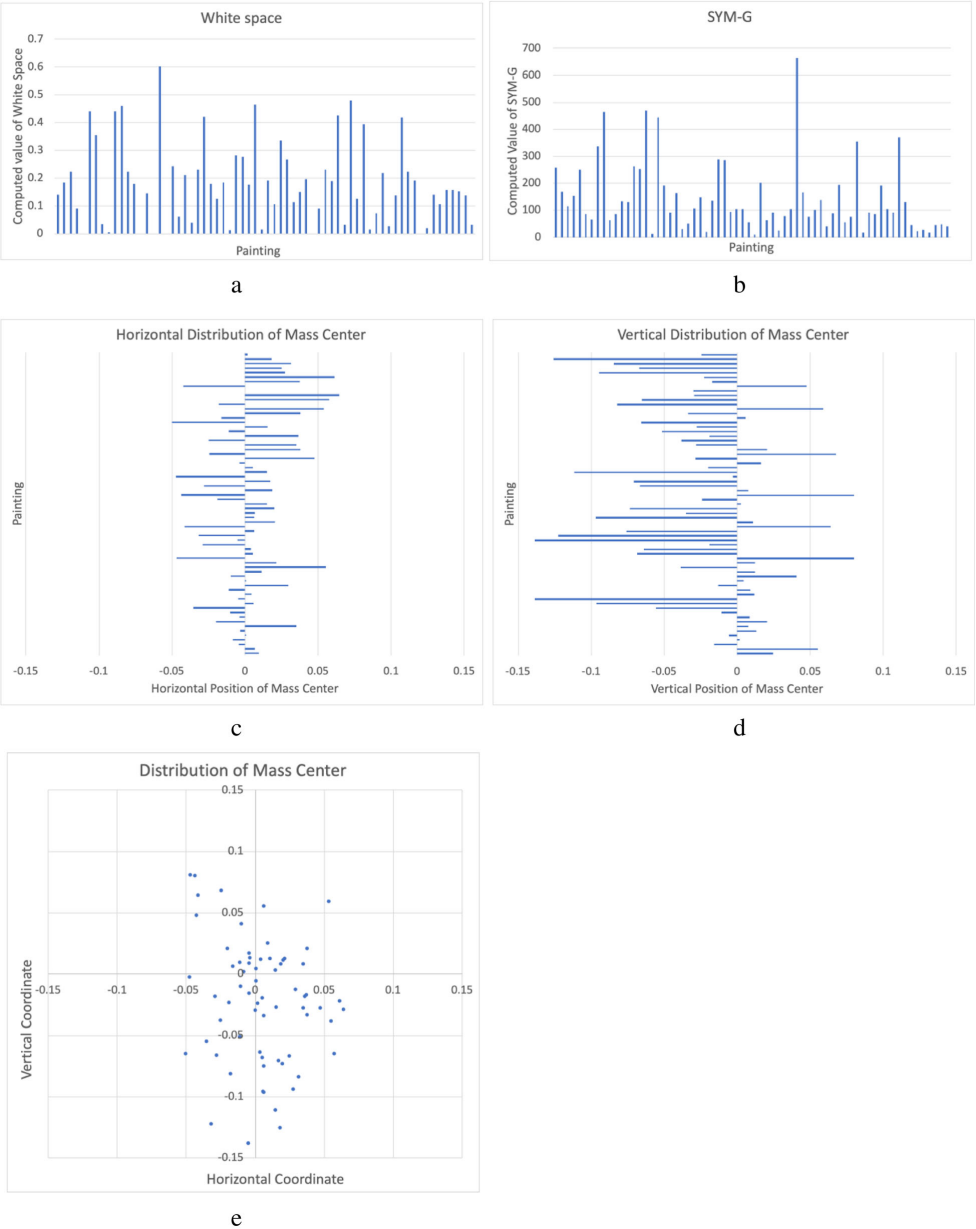
The distribution of white space (**a**), SYM-G (**b**), horizontal coordinates (**c**) and vertical coordinates (**d**) of the mass center. The distributions of the relative coordinates of the mass centers on the paintings

The results of Coefficients in Table 1 indicate that the percentage of white space positively influences the visual order (*Regression Coefficients* = 0.234). This implies that a painting with a higher percentage of white space tends to be more ordered. Serving as a rhetoric figure, white space is deliberately left by the artists to convey information [33], being the simplest sensory input yet rich in information. When successfully recognized and interpreted as intended by viewers, white space is considered meaningful. This interpretation can lead to a sense of order in aesthetic processing. The interpretation of the visual information contained in white space was thought to be difficult to process but turned out to be easy. The viewers successfully recognize the sky, window, river from the white spaces. The difficulty in interpretation turns out to be pleasure in information processing [2]. This ease in interpretation of white space makes viewers feel that the painting is ordered. Figure 3a shows the distribution of white space values.

Mass center’s distance to the image center positively influences the visual order and the Manhattan distance taking into account the size of each painting is more significant than other distance features. A painting not balanced in the middle is more ordered than the one balanced in the exact middle. Only considering mass center’s distance to the image center, we may think that, the more off-center, the more orderly is the painting. But statistics of the mean and standard deviation of mass center’s distance to center in Table 2 imply that most of the paintings’ mass centers are close to the paintings’ physical centers. We visualize the distributions of relative coordinates of mass centers in Fig. 3e and find that mass centers are not far away from the image centers. We define the center of a painting as 0 while − 0.5 and 0.5 at the extreme edges (left and right, bottom and top). Figure 3c shows that the horizontal coordinates are between − 0.05 and 0.1, near the middle, without preference of left or right. The vertical coordinates are more off-center than the horizontal coordinates and tend to be more biased toward the bottom (Fig. 3d). But this preference does not have any correlation to the visual order of the paintings.

Symmetry is an interesting feature representing order and law [34]. Symmetry and beauty are often claimed to be related. Symmetry in aesthetics is a term in a global scale. But Chinese ink paintings are usually not composed in an explicit global symmetry and symmetry is typically seen at a local scale. Local symmetry calculates the difference in the local scale [28]. A lower value indicates greater local symmetry while a higher value means lower local symmetry. Table 1 shows that SYM-G negatively influences the rating score of visual order (Regression Coefficients = − 0.311). This means SYM-G can increase the visual order of the paintings. In other words, if the painting is more locally symmetrical, it is more ordered. SYM-I and SYM-G are both correlated to visual order as shown in Table 3. But the latter is more significant than the former. The distribution of SYM-G values is shown in Fig. 3b.

Except these three features, the correlation results in Table 3 show that image segments related features do not show any significant relation to visual order, and *x*-axis of mass center appears to be relevant to visual order. But as Fig. 3 visualizes, most of mass centers of paintings are not far away from the image centers and relative *x*-axis of mass center also has a low Pearson correlation value. Compared with the horizontal position of the mass center, the vertical position has a clear influence on visual order.

To test the prediction power of regression, we also use 80% of the painting dataset as the training set and the remaining part as the test set. We obtain the R^2^ (coefficient of determination) equaling 0.41 and mean square error equaling 0.22.

## Conclusions

Based on the previous research on aesthetic quality assessment and art theory, our study proposes measurements for several visual features influencing the visual order of Chinese ink paintings and provides an objective view in understanding human aesthetic assessment on the paintings. By extracting visual features and generating regression models, we find that the mass center’s distance to the image center, local symmetry and white space can together influence the visual order.

White space is essential in the study of Chinese ink paintings. It is widely used in many well-known masterpieces and depicts vast, formless objects, such as clouds, rivers, lakes, deeply rooted in Chinese philosophy and aesthetics. The classical Taoism literature highlights the concepts of the emptiness and regards white space as of equal importance to colored space [35]. White space is the simplest form of sensory input, as a stylistic element that lacks transparent semantic meanings but can convey a simple but imaginative feel. It also serves a visual rhetoric figure with an implicit yet clearly defined meaning. When viewers recognize the contents of a white space, they could possibly recognize the artist’s intentional design. This recognition process could be transformed into an aesthetic appreciation of order. Our previous experiment shows that all the significant white spaces have successfully conveyed intended meanings.

Existing literature supports that symmetry means something well-proportioned, well-balanced, and denotes the concordance of several parts [36]. Our experiment on the Chinese ink paintings also supports this statement. The elaborate design of local symmetry in Chinese ink paintings brings people an arranged and ordered appreciation.

Researchers give various interpretations of the balance point in a picture, e.g., around the mid-point or Golden Section ratio. In our experiment, the balance points of Chinese ink paintings are clustered around the mid-point. The relative balance on *y*-axis is more varied and off-center than the balance on the *x*-axis. The artists intentionally or unconsciously make the mass centers below the center of paintings. This tendency does not influence the viewer’s perceived order, though the participants prefer an off-center balance point, which potentially increases the visual order.

Artistic feelings could be drastically different among people of all walks of life, ranging from complete novices to professionally trained artists. Their perception of visual order could also vary significantly. This research only collects ratings from ordinary people. In terms of genres of paintings and different cultures and religions, our work is limited to contemporary Chinese ink paintings. We are curious about whether we could repeat our findings, such as that local symmetry increasing visual order, on other styles or genres of paintings. For example, existing literature supports that pure symmetry is too harsh, rigid and unlifelike [36, 37]. Symmetry is formal rigidity and constraint while asymmetry is playing and freedom [34]. It is possible that symmetry or even local symmetry is not always preferred in other genres of artworks. Also, we use a linear regression model in this study and find many features are correlated. In our future work, we should apply a dimension reduction technique, such as PCA, to reduce dimensionality on the entire feature space [38, 39].

The exploration of visual order in a quantitative fashion is our long-term goal. We aim at eventually being able to comprehensively assess paintings’ visual order. The aesthetic assessment of various genres of paintings is an interdisciplinary topic involving computing, visual arts, psychology and philosophy. To generalize a robust model in assessing visual order, or even beauty, of a wide range of artworks, one needs to take into account many other variables and conduct much more comprehensive experiments, which is a tremendous challenge. The huge differences, variety of artworks and cultural preferences make the challenge even harder, that need to be tackled step by step.

## Data Availability

No applicable.

## Abbreviations

FLS: First largest segment
SLS: Second largest segment
SYM-I: Local symmetry using intensity
SYM-G: Local symmetry using gradient
M: Measure
O: Order
C: Complexity
CCM: Color complexity measurement
